# Peptide hormone ELABELA promotes rat bone marrow-derived mesenchymal stem cell proliferation and migration by manipulating the cell cycle through the PI3K/AKT pathway under the hypoxia and ischemia microenvironmemt

**DOI:** 10.1186/s13287-021-02691-1

**Published:** 2022-01-28

**Authors:** Xuxiang Chen, Changqing Zhou, Daishi Xu, Xin Liu, Shuangmei Li, Jingyu Hou, Kanglong Zhang, Chaotao Zeng, Guanghui Zheng, Haidong Wu, Hao Wu, Wuming Wang, Jiaying Fu, Tong Wang

**Affiliations:** 1 Department of Emergency, the Eighth Affiliated Hospital of Sun Yat-sen University, Shenzhen, 518003 Guangdong People’s Republic of China; 2 Department of Emergency, the Sun Yat-sen Memorial Hospital of Sun Yat-sen University, Guangzhou, 510120 Guangdong People’s Republic of China

**Keywords:** ELABELA, Rat bone marrow-derived mesenchymal stem cells, Proliferation, Migration, Cell cycle

## Abstract

**Background:**

Mesenchymal stem cells (MSCs) are emerging as a potential candidate for stem cell transplantation to repair myocardial tissue in myocardial infarctions (MI). However, there are some pivotal limitations such as poor survival and low migration capacity of MSCs in hypoxic and ischemic microenvironments of MI. Our previous work verified that ELABELA (also abbreviated as ELA), a peptide hormone, could play a role as a growth factor and prolong the life span of rat bone marrow-derived mesenchymal stem cells (RAT BM-MSCs) under hypoxic and ischemic conditions. Nevertheless, the influence of ELA on the cell cycle, proliferation, and migration remains elusive. This study will further explore the improvement of the biological functions of ELA-treated RAT BM-MSCs, so as to provide a reference for improving the efficacy of RAT BM-MSCs in MI.

**Methods:**

Rat BM-MSCs were isolated from 80 to 120 g Sprague Dawley rats by flushing femurs and tibias under the aseptic condition. RAT BM-MSCs of the third passage were divided into control group, hypoxic/ischemic (H/I) group, ELA group, ELA-LY group and LY group. RAT BM-MSCs were cultured under normoxia in control group. In H/I group, RAT BM-MSCs were exposed to hypoxia (1% O2) and serum deprivation for 24 h. RAT BM-MSCs in ELA group were treated with 5 µM ELA prior to the H/I exposure for 24 h. The PI3K/AKT inhibitor, LY294002 (50 µM), was used in ELA-LY group and LY group to observe the effect of ELA on PI3K/AKT activation. Cell proliferation ability was examined by CCK-8. Cell cycle was assessed with flow cytometry. Cell migration was evaluated by Transwell assay. Expression levels of total-AKT, phosphorylated-AKT, and cell cycle-associated proteins were examined by Western blotting.

**Results:**

ELA-treated RAT BM-MSCs exhibited significantly higher proliferation ability, cell viability, and migration under H/I conditions. The cell cycle analysis showed that an increased proportion of cells in the S and G2/M phases of the cell cycle were observed in ELA-treated RAT BM-MSCs. The addition of ELA activated the PI3K/AKT signaling pathway. Additionally, upon treating with the inhibitor of the PI3K/AKT signaling pathway, ELA-triggered proliferation, cell viability, and migration were abrogated.

**Conclusions:**

ELA can be used to enhance the proliferation ability, cell viability, and migration of RAT BM-MSCs through the PI3K/AKT signaling pathway and alleviate cell cycle arrest at the G0/G1 phase under hypoxic and ischemic injury. Thus, this study provides a promising strategy that ELA may help to optimize the mesenchymal stem cell-based therapy in MI.

## Background

Mesenchymal stem cells (MSCs) are commonly considered as a potential cell type in regenerative medicine due to their multiple differentiation, proliferation, and homing capacity [1]. MSCs have shown promising treatment potential for damaged cardiac tissue and improvement of cardiac function in myocardial infarction (MI) [2, 3]. However, the curative effect of MSCs in MI is influenced by many aspects including the low survival rate of MSCs and insufficient number of MSCs migrating into injured cardiac sites under hypoxic and ischemic microenvironments [4, 5]. Hence, how to improve MSCs proliferation, cell viability, and migration under the hypoxia and ischemia microenvironment of MI is a question that needs further researches.

Numerous studies have shown that cell proliferation and the cell delivery process of MSCs can be affected by chemical factors such as cytokines, growth factors, and chemokines [6–8]. ELABELA (also abbreviated as ELA) is a newly discovered bioactive peptide that both exerts protective effects on the self-renewal capacity and increases resistance to oxidative stress injury in human embryonic stem cells (hESCs) [9, 10]. The ELA-APJ axis is a promising therapeutic target for cardiovascular diseases [11, 12]. Intriguingly, our previous study found that ELA could alleviate the apoptotic level of MSCs via the PI3K/AKT and ERK1/2 signaling pathways under hypoxic and ischemic conditions [13]. The PI3K/AKT signaling pathway is known to play a central role in modulating cellular processes of MSCs involving cell proliferation, migration, and apoptosis [14, 15]. Besides, the researchers have found that PI3K/AKT can accelerate cell proliferation through the alteration of cell cycle progression [16, 17]. The cell cycle is commonly divided into five phases: G0, G1, S, G2, and the M phase. In eukaryotic cells, the protein levels of cyclins vary synchronously according to the cell cycle progression. Among the 11 members of the cyclin family, Cyclin D1 and Cyclin E are two key factors that can promote G1-phase transition and S-phase entry [18, 19]. However, whether ELA promotes cell proliferation and migration of MSCs through the PI3K/AKT signaling pathway and modulation of cell cycle progression remains unknown.

In this study, we expanded our previous study by investigating ELA’s effects on rat bone marrow-derived mesenchymal stem cells (RAT BM-MSCs) proliferation and migration in hypoxic and ischemic conditions and clarifying the positive function of ELA on RAT BM-MSCs via the PI3K/AKT/CyclinD1/E signaling pathway.

## Methods

### Ethics statement

The ethical committee at Sun Yat-sen University approved all the procedures on rats in this study (approval number: 2019-057-01). Sprague Dawley rats (SD rats, male, weighed 100 ± 20 g, SPF grade) were purchased from the Animal Experimental Center of Sun Yat-sen University.

### Chemicals

ELA with peptide fragments of 32 amino acids (sequence: QRPVNLTMRRKLRKHNCLQRRCMPLHSRVPFP) was synthesized by GL Biochem Shanghai Ltd (China). The ELA powder was at 96.08% purity and stored at − 20 °C. The ELA was dissolved and diluted with phosphate-buffered solution (PBS) to 5 µM and then sterilized with 0.22 µm filters before each use.

### Isolation and culture of RAT BM-MSCs

RAT BM-MSCs were obtained from SD rats by flushing femurs and tibias as previously described [13, 20]. The isolated RAT BM-MSCs were cultured in the complete culture medium which was composed of low-glucose Dulbecco modified eagle medium (GIBCO), 10% fetal bovine serum (GIBCO), penicillin (100 IU/ml) and streptomycin (100 μg/ml) (HyClone). According to the bone-marrow adherent methodology, after 3 days, the medium was replaced with fresh medium to remove the non-adherent cells, and the medium was replaced every 2–3 days before cells gained 90% confluence. Fluorescence-activated cell sorting (FACS) was used to identify the cell markers of RAT BM-MSCs as previously reported [13, 20]. The third-passaged RAT BM-MSCs with positive expression of CD44 and CD29, but negative expression of CD34, were used to perform the following experiments, which are consistent with other studies [21, 22].

### Hypoxia and ischemia (H/I) treatment of RAT BM-MSCs

The cells cultured in normoxia (20% O_2_) was a negative control. Hypoxia and ischemia (H/I) group-cells were incubated in serum-free and hypoxia (1% O_2_) conditions in a hypoxia incubator chamber for 24 h as previously described [13]. The serum-free medium contains low-glucose Dulbecco modified eagle medium, penicillin (100 IU/ml) and streptomycin (100 μg/ml), without fetal bovine serum. ELA group-cells were stimulated with ELA at a concentration of 5 μM for 12 h prior to H/I exposure for 24 h. ELA + LY group-cells were treated with 50 μM LY294002 (PI3K/AKT pathway inhibitor) for 2 h prior to the ELA, and then, the cells were exposed to the H/I conditions for 24 h. LY group-cells were treated with 50 μM LY294002 for 2 h prior to H/I exposure for 24 h. After the above treatments, cells in each group will be used instantly in subsequent experiments.

### Cell proliferation and viability assay

Cell Counting Kit-8 (CCK-8 Kit) was used to examine cell proliferation and viability of RAT BM-MSCs under exposure to nomoxia and H/I conditions for 0 h, 24 h, 48 h and 72 h. RAT BM-MSCs were plated into 96-well plates and incubated with 10 μl CCK8 for 2 h. The optical density values (OD450) were measured by a micro-plate reader.

### Cell migration assay

Cell migration assay was performed using Transwell chambers with 8-μm pore size (Corning). RAT BM-MSCs were starved in serum-free medium containing low-glucose Dulbecco modified eagle medium, penicillin (100 IU/ml) and streptomycin (100 μg/ml) for 12 h and then seeded into the upper chamber at the density of 6 × 10^4^ cells with 100 μl serum-free medium. Subsequently, 600 μl complete culture medium was added into the lower chamber and incubated with the upper chamber in a 37 °C incubator for 10 h. Then, the upper chamber was removed and cotton-tipped applicators were used to carefully remove the remaining cells that have not migrated to the bottom of the membrane. Additionally, cells on the upper chamber were fixed with 4% paraformaldehyde for 15 min. After the membrane of the upper chamber was dry, the upper chambers were placed into 0.1% crystal violet dyes to stain cells for 20 min at room temperature. Five random micrographs per group were viewed under an inverted microscope, and the quantitative data analysis was performed with ImageJ.

### Cell cycle analysis

RAT BM-MSCs were cultured in serum-free medium overnight to obtain a synchronous cell cycle before treatment. Cell cycle was analyzed with Cell Cycle Detection Kit (Keygen Biotech). RAT BM-MSCs were collected and washed with prechilled PBS three times. RAT BM-MSCs were then fixed in 70% cold ethanol at 4 °C overnight. Subsequently, RAT BM-MSCs were washed with prechilled PBS and stained with 500 μl propidium iodide (PI)/RNase at room temperature for 30 min in the dark. Finally, cell cycle analysis was performed with flow cytometer (BD LSRFortessa).

### Western blotting

After the designated treatment, cells were washed with cold PBS twice and lysed with RIPA lysis buffer (Beyotime) containing protease and phosphatase inhibitor cocktail (CWBIO) for 30 min on ice. The protein concentration was quantified with a bicinchoninic acid (BCA) assay kit (CWBIO). After mixing with SDS sample loading buffer, the protein in each group was heated at 100 °C for 10 min. Equal amounts of proteins were loaded into 10% sodium dodecyl sulfate–polyacrylamide gel electrophoresis (SDS-PAGE) and were transferred to polyvinylidene difluoride (PVDF) membranes. Subsequently, the membranes were blocked with blocking solution (5% fat-free milk in 1 × TBST) for 1 h and incubated with anti-rabbit primary antibodies at 4 °C overnight. The primary antibodies: AKT (1:1000; #4691), phospho-Akt (Ser473) (1:2000; # 4060), Cyclin D1 (1:1000; #2978 s), and GAPDH (1:1000; #2118) were purchased from Cell Signaling Technology; CyclinE (1:600; #sc-377100) was purchased from Santa Cruz Biotechnology. After rinsing with 1 × TBST three times, the membranes were incubated with anti-rabbit IgG and HRP-linked antibody (1:2000, Cell Signaling Technology) at room temperature for 1 h. The membranes were washed three times with 1 × TBST for 10 min each and then detected using a ChemiDoc™ Touch Imaging System (Bio-Rad).

### Statistical analysis

Data were expressed as mean ± standard deviation. Each experiment was performed in triplicate. Statistical comparisons among groups were detected using analysis of variance (ANOVA) with a Bonferroni post hoc test. *P* < 0.05 was considered statistically significant.

## Results

### ELA enhanced RAT BM-MSCs proliferation under hypoxic-ischemic injury

**Fig. 1:**
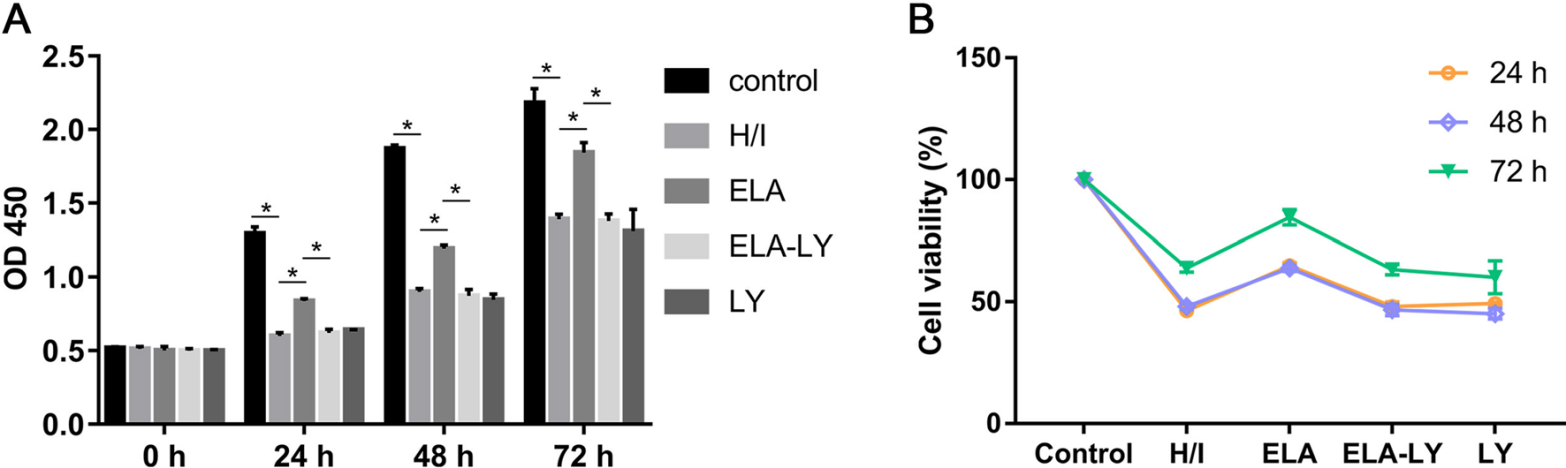
ELA promotes RAT BM-MSCs proliferation (**A**) and cell viability (**B**) and PI3K/AKT is required in this effect under H/I injury at 0 h, 24 h, 48 h and 72 h. The treatment time of ELA (at a concentration of 5 μM) was 12 h prior to H/I injury. *P < 0.05. ELA, ELABELA; RAT BM-MSCs, Rat bone marrow-derived mesenchymal stem cells; H/I, hypoxic-ischemic; LY, LY294002

**Fig. 2:**
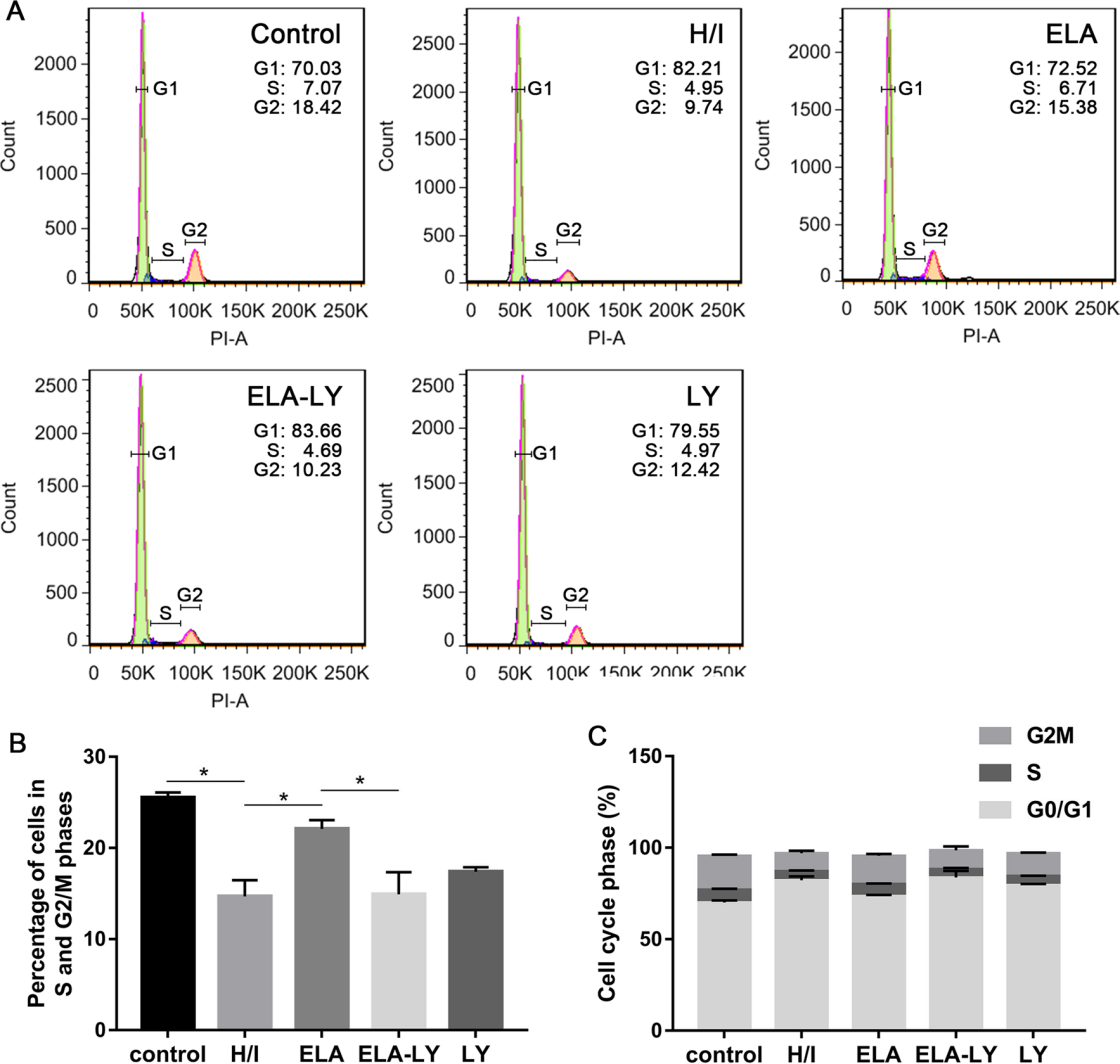
The impact of ELA on the cell cycle progression under H/I conditions for 24 h. The treatment time of ELA (at a concentration of 5 μM) was 12 h prior to H/I injury. **A** The representative results of the cell cycle. **B** The percentage of S and G2/M phases of RAT BM-MSCs. **C** The percentage of cell cycle distributions of RAT BM-MSCs involving G0/G1, S, and G2/M. *P < 0.05. ELA, ELABELA; RAT BM-MSCs, Rat bone marrow-derived mesenchymal stem cells; H/I, hypoxic-ischemic; LY, LY294002

RAT BM-MSCs treated with 5 μM ELA displayed increased proliferation capacity (*P* < 0.05, Fig. 1A) and cell viability (*P* < 0.05, Fig. 1B) when compared with the H/I group under hypoxic and ischemic conditions at 0 h, 24 h, 48 h and 72 h. These results indicated that ELA enhanced RAT BM-MSCs proliferation under hypoxic-ischemic injury in vitro. The cell cycle analysis revealed that ELA promoted a higher percentage of S and G2/M phase cells compared with H/I group (*P* < 0.05, Fig. 2A–C), and it is consistent with the result in Fig. 1A. To probe the role of the PI3K/AKT signaling pathway in ELA-promoted RAT BM-MSCs proliferation, cells were cultured in medium with a PI3K/AKT inhibitor (LY294002) for 2 h prior to ELA before H/I exposure. Compared with ELA group, the proliferation capacity (*P* < 0.05, Fig. 1A) and cell viability (*P* < 0.05, Fig. 1B) of RAT BM-MSCs in ELA-LY group were significantly decreased at different time points, indicating that LY294002 counteracted the role of ELA on RAT BM-MSCs proliferation. Besides, we found that the percentage of S- and G2/M-phase cells in ELA-LY group dramatically decreased in comparison with ELA group (*P* < 0.05, Fig. 2B, C). This suggested that ELA may promote RAT BM-MSCs proliferation via the PI3K/AKT pathway and regulation of cell cycle progression.

### ELA promoted RAT BM-MSCs migration under hypoxic-ischemic conditions

**Fig. 3:**
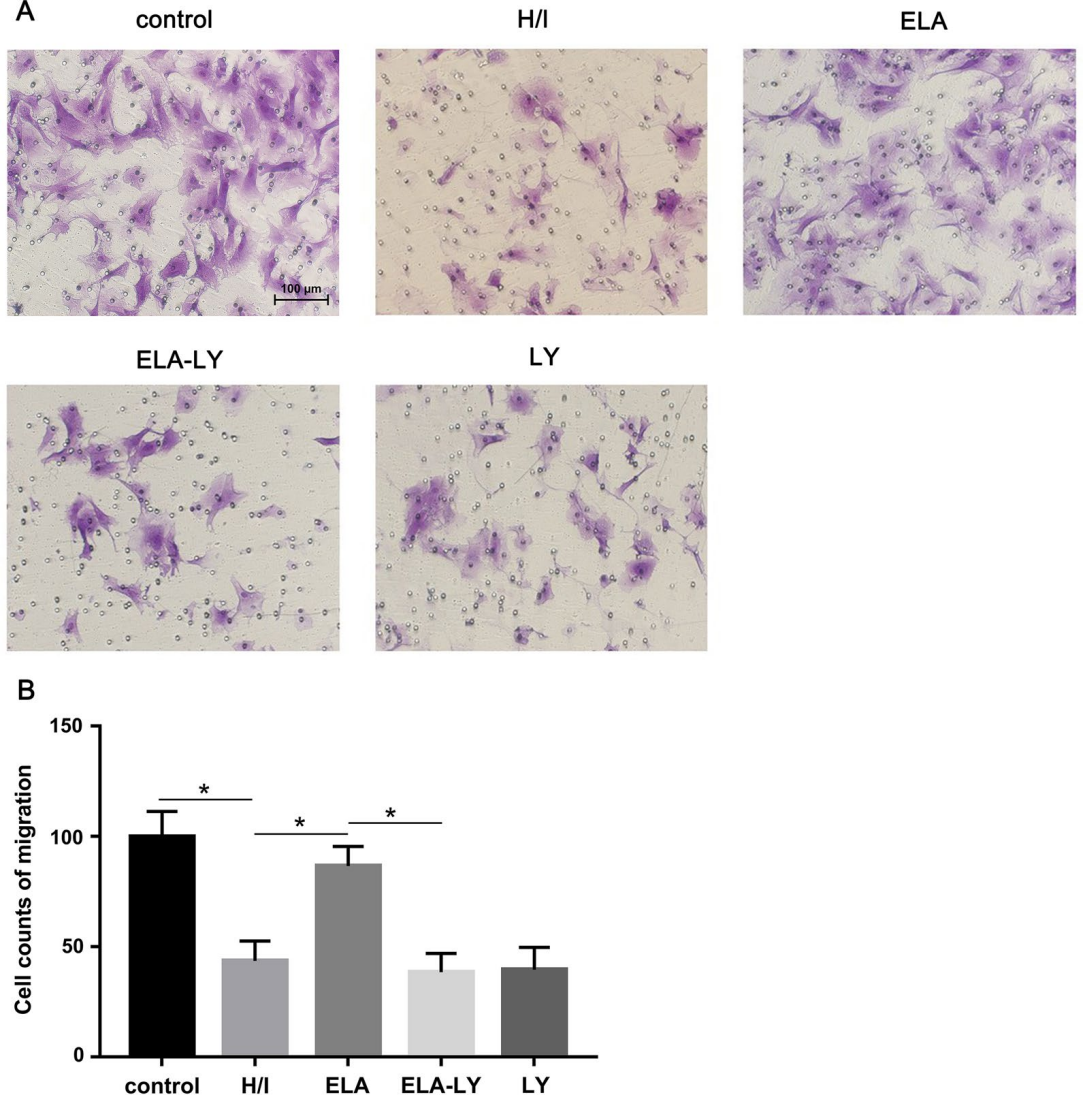
ELA promotes RAT BM-MSCs migration within 10 h of H/I injury. The treatment time of ELA (at a concentration of 5 μM) was 12 h prior to H/I injury. **A** The representative figures in different experimental groups (Scale bar = 100 μm). **B** Cell counts of RAT BM-MSCs migration. *P < 0.05. ELA, ELABELA; RAT BM-MSCs, Rat bone marrow-derived mesenchymal stem cells; H/I, hypoxic-ischemic; LY, LY294002

Cell migration analysis showed a higher capacity of cell migration in ELA group in comparison with H/I group under hypoxic-ischemic conditions (*P* < 0.05, Fig. 3A, B). We hypothesized that ELA might promote RAT BM-MSCs migration by activating the PI3K/AKT signaling pathways. To investigate this question, we culture the RAT BM-MSCs in medium with LY294002 for 2 h prior to ELA. We demonstrated that suppression of the PI3K/AKT signaling pathways counteracted the role of ELA in promoting RAT BM-MSCs migration (Fig. 3A, B), indicating that ELA may have promoted RAT BM-MSCs migration via the PI3K/AKT pathway.

### Mechanism of ELA’s roles in modulating RAT BM-MSCs proliferation and migration

**Fig. 4:**
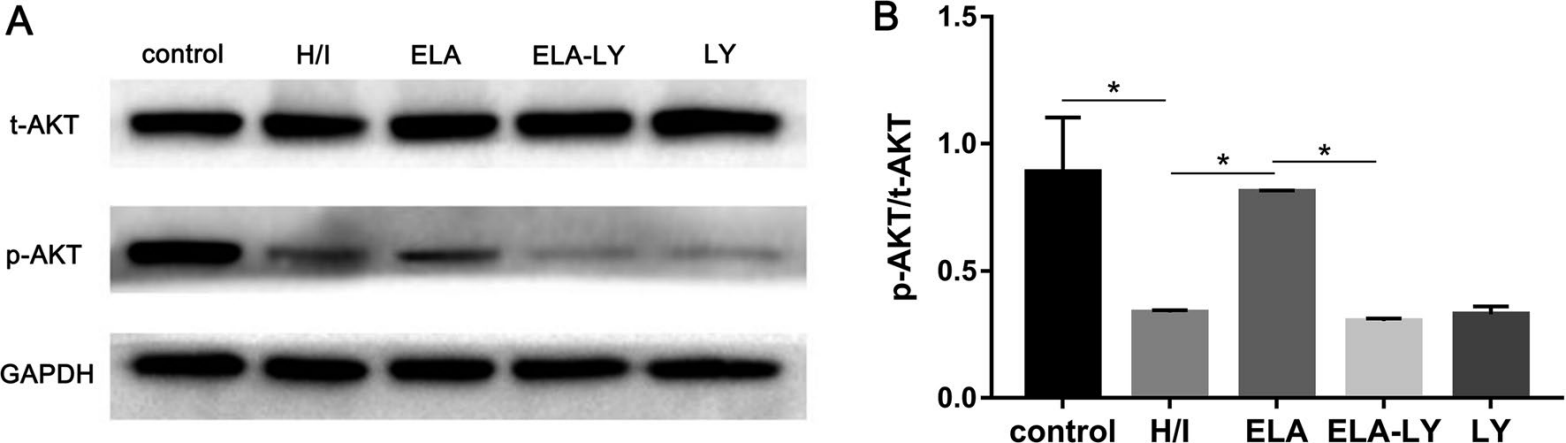
ELA activates the PI3K/AKT signaling pathway in RAT BM-MSCs under H/I conditions for 24 h. The treatment time of ELA (at a concentration of 5 μM) was 12 h prior to H/I injury. **A** The representative blots showed the protein level of total-AKT and p-AKT (Ser 473) in RAT BM-MSCs. **B** The quantitative data were calculated with the ratio of p-AKT and t-AKT. *P < 0.05. ELA, ELABELA; RAT BM-MSCs, Rat bone marrow-derived mesenchymal stem cells; H/I, hypoxic-ischemic; LY, LY294002; t-AKT, total-AKT; p-AKT, phosphorated-AKT

**Fig. 5:**
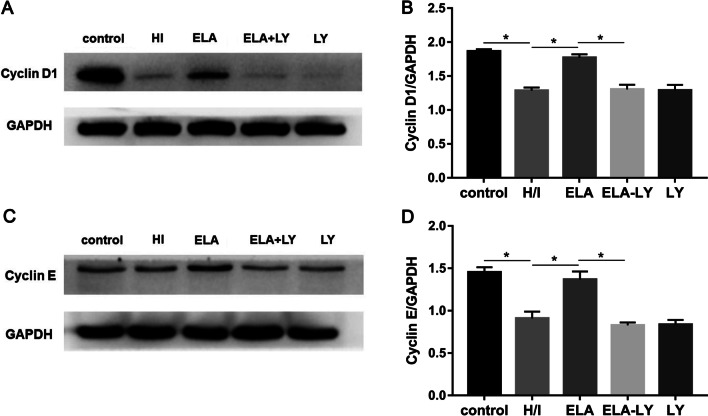
ELA modulates the cell cycle-related proteins Cyclin D1 and Cyclin E in RAT BM-MSCs under H/I conditions for 24 h. The treatment time of ELA (at a concentration of 5 μM) was 12 h prior to H/I injury. **A**, **C** The protein levels of Cyclin D1 and Cyclin E in different groups. **B**, **D** The quantitative data were calculated with the ratio of Cyclin D1/GAPDH and Cyclin E/GAPDH. *P < 0.05. ELA, ELABELA; RAT BM-MSCs, Rat bone marrow-derived mesenchymal stem cells; H/I, hypoxic-ischemic; LY, LY294002

To explore the regulation mechanism of ELA promoting RAT BM-MSCs proliferation and migration, we examined the protein level of phospho-AKT, total-AKT, Cyclin D1, and Cyclin E. The protein expression ratio of phosphor-AKT and total-AKT were significantly increased in ELA group compared with H/I group under hypoxic-ischemic microenvironment (*P* < 0.05, Fig. 4A, B), but there was no significant difference between the ELA-LY and LY groups (Fig. 4A, B). Additionally, Cyclin D1 and Cyclin E both contributed to the G1/S phase progression. Compared with the H/I group, the expression levels of Cyclin D1 and Cyclin E were significantly increased in the ELA group (*P* < 0.05, Fig. 5A, B; *P* < 0.05, Fig. 5C, D), and the PI3K/AKT inhibitor LY294002 could potently reverse this trend (*P* < 0.05, Fig. 5A–D). These results indicated that ELA promoted RAT BM-MSCs proliferation and migration through the activation of PI3K/AKT signaling pathway and up-regulation of the cell cycle modulators including Cyclin D1 and Cyclin E.

## Discussion

In the present study, ELA was discovered to be a positive regulator in RAT BM-MSCs proliferation and migration under hypoxic and ischemic injury. Moreover, these effects were correlated with the activation of the PI3K/AKT signaling pathway and the alteration of cell cycle progression. To our knowledge, this is the first study to reveal the beneficial influences and the potential mechanisms of ELA on RAT BM-MSCs proliferation and migration under hypoxic and ischemic injury.

Currently, the therapeutic efficacy of stem cell-based treatment in MI is still insufficient [23, 24]. The ischemic and hypoxic microenvironment caused by MI will cause decreased cell proliferation ability and homing efficiency of MSCs [25]. An extremely limited number of MSCs were able to reach the damaged cardiac tissue and exert therapeutic effects after transplantation [26, 27]. Additionally, the transplanted MSCs showed poor proliferation capacity and unsatisfactory cell viability to persist in the ischemic myocardial tissues [28]. Hence, MSCs proliferation and migration ability should be enhanced with feasible interventions to maximize their therapeutic potential in cardiac repair.

ELA can serve as an endogenous hormone which is required for normal heart and vasculature formation [29, 30]. ELA also has a role in regulating endodermal cell migration [31]. In addition, it has been mentioned that ELA transcript exists in human pluripotent stem cells and ELA can enhance the self-renewal of hESCs [32, 33]. However, the biological functions of ELA in RAT BM-MSCs under H/I conditions remain unknown.

Our results found that ELA at the concentration of 5 μM significantly promote the RAT BM-MSCs proliferation, cell viability, and migration under H/I conditions. Reduced proliferative capacity under H/I exposure might be due to cell cycle arrest [34]. To address this issue, we further performed cell cycle analysis using flow cytometry. A decreased percentage of RAT BM-MSCs in S and G2/M phases of the cell cycle could be observed under H/I exposure. Contrarily, the number of ELA-treated RAT BM-MSCs in S and G2/M phases of the cell cycle increased under H/I exposure. The regulation mechanism of ELA on the cell cycle progression in RAT BM-MSCs is consistent with that of ELA in hESCs. Lena Ho et al. found that ELA promotes hESC self-renewal by regulating cell cycle progression showing a decreased cell proportion in the G1 phase and an increased cell proportion in S, G2, and M phases [10]. We sought to investigate the necessity of the PI3K/AKT pathway in the control of cell-cycle progression, because the PI3K/AKT pathway has been known to regulate the G1/S conversion.

We confirmed this result by using LY294002, a specific inhibitor of PI3K/AKT, to block the AKT activation and found that LY294002 could potently attenuate the motivating effect of ELA on the proliferation and migration of RAT BM-MSCs. Concomitantly, the phosphorylation of AKT was retained in ELA-treated RAT BM-MSCs under H/I injury, suggesting that ELA-treatment activated AKT and promoted the RAT BM-MSCs proliferation and migration.

The PI3K/AKT signaling pathway is an essential node in RAT BM-MSCs, and AKT activity is involved in modulating RAT BM-MSCs proliferation, migration, and angiogenesis, among other processes [35, 36]. In addition, ELA has been confirmed to promote cell proliferation and cell viability through AKT activation in other cell types such as hESCs [10], umbilical vein endothelial cells, [37] and BeWo cells [38]. AKT is the primary mediator of PI3K, and a few downstream substrates of AKT may control cell cycle progression [39]. Our study showed that G1/S transition might be the potential target of anti-proliferation of RAT BM-MSCs under H/I injury. Thus, we focused on the key regulators of G1-phase cyclins downstream of PI3K/AKT. Our results showed that Cyclin D1 and Cyclin E were repressed in RAT BM-MSCs under H/I injury and were consistent with the cell cycle results. In contrast, ELA promoted RAT BM-MSCs proliferation by regulating Cyclin D1 and Cyclin E, while LY294002 counteracted these effects. One reason for these results might be that ELA alleviated the G1/S cell cycle arrest caused by the H/I microenvironments in RAT BM-MSCs through PI3K/AKT signaling pathway which regulated Cyclin D1 and Cyclin E.

Apart from cell proliferation, our study illustrated the beneficial effect of ELA on promoting RAT BM-MSCs migration in the H/I microenvironment. Meanwhile, we explored the regulatory mechanisms of ELA-induced migration of RAT BM-MSCs. It is well known that the PI3K/AKT signaling pathway plays an important role in regulating cell motility and actin cytoskeleton [40]. Furthermore, we inhibited the PI3K/AKT pathway in RAT BM-MSCs with LY294002 for 2 h prior to ELA. The results showed no improvement of cell migration in RAT BM-MSCs treated with LY294002 plus ELA under H/I injury. In addition, we verified that the AKT activation is essential for the promoting effect of ELA on RAT BM-MSCs migration.

There were two limitations in our study. Although AKT activation was involved in the cell migration of ELA-treated RAT BM-MSCs, how ELA regulates the downstream molecules remains unknown. Another limitation of our study was that we did not monitor the function and mechanisms of ELA on RAT BM-MSCs proliferation and homing ability in vivo, and further study is needed to verify the therapeutic effect and regulatory mechanisms of ELA-treated RAT BM-MSCs in rat models of MI from pharmacological and pathophysiological perspectives.

## Conclusions

In conclusion, we provided solid evidence that ELA treatment enhances the RAT BM-MSCs proliferation and migration capacity in H/I microenvironments in vitro through the activation of the PI3K/AKT signaling pathway. We also demonstrated that ELA promoted the G1/S transition of cell cycle and upregulated cyclins including Cyclin D1 and Cyclin E.

## Data Availability

All data generated or analyzed during this study are included in this published article.

## Abbreviation

RAT BM-MSCs: Rat bone marrow-derived mesenchymal stem cells
MSCs: Mesenchymal stem cells
ELA: ELABELA
PI3K: Phosphatidylinositol 3 kinase
p-Akt: Phosphorylated Akt
t-AKT: Total AKT
CCK8: Cell counting kit-8
H/I: Hypoxic and ischemic
LY: LY294002
PVDF: Polyvinylidene difluoride
SDS–PAGE: Sodium dodecyl sulfate–polyacrylamide gel electrophoresis
PBS: Phosphate-buffered solution
SD: Sprague–Dawley
